# Pain management among medical in-patients in Blantyre, Malawi

**DOI:** 10.1186/1755-7682-2-6

**Published:** 2009-03-26

**Authors:** Adamson S Muula, Humphreys E Misiri

**Affiliations:** 1 Department of Public Health, Division of Community Health, College of Medicine, University of Malawi, Blantyre, Malawi

## Abstract

**Background:**

Pain is a leading symptom which influences patients to seek medical attention. The management of pain among patients attending in-patient care in southern African countries has been little described. Information regarding the prevalence of pain and the quality of its management may be useful in guiding clinical decisions, training of health workers and health care quality improvements.

**Methods:**

A hospital-based audit was conducted to estimate the prevalence of pain and examine the quality of its management among patients admitted to adult medical wards at Queen Elizabeth Central Hospital (QECH), Blantyre, Malawi in 2004. Data were abstracted from ward charts of consecutive patients' who had been either been discharged or had died within a specified period. Characteristics of interest included; socio-demographic data, presence or absence of pain at admission, characterization or description of pain when present, and drug treatment given. Data were analyzed to obtain frequencies and proportions of the characteristics and assess the prevalence of pain and quality of care.

**Results:**

A total of 121 patients' case notes were reviewed and the prevalence of pain was recorded for 91 (75.2%) of the patients. Clinicians had recorded pertinent information regarding pain management with the following frequency: pain severity or intensity 5/91 (5.5%), alleviating factors 5 (5.5%), pain radiation 7 (7.7%), exacerbating factors in 9 (9.9%) and periodicity in 43 (47.3%) of the cases. Males with pain were more than 3 times more likely to receive analgesic as compared to females, p < 0.01. Paracetamol was the commonest analgesic prescribed.

**Conclusion:**

Inadequate management of pain among patients attending medical wards at QECH was found. There is need for prospective studies to further characterize pain management and identify pain management gaps in Malawi. Interviews of clinicians and documentation of observations within clinical practice are likely to be of value.

## Background

Pain is a common medical symptom prompting patients to seek care. However adequate management of pain remains a global challenge. In 1982, the World Health Organization (WHO) recognized inadequate management of (cancer) pain as an international health problem [1]. While cancer pain management has certainly received some attention however, pain experienced during childbirth, that arising from infectious diseases, in post-surgical care and in other clinical situations remains under-appreciated among researchers in southern Africa. In addition, while there is deserved interest in the clinical management of tuberculosis, malaria and AIDS (acquired immune-deficiency syndrome) in resource-limited settings of Southern Africa, little attention has been made to the immediate and long-term alleviation of painful syndromes that may be associated with these conditions.

Pain may be acceptable to patients as an inevitable and natural aspect of illness; this may affect patients' tolerance of the symptom (pain) and request for analgesia [2]. Health professionals may also accept pain as inevitable and expect patients to "bear it up." Patients may also hesitate to bring up the discussion of their pains with their care providers for a variety of reasons, including lack of effective communication skills and the fear of medications [3-5]. This however, does not mean that patients are not concerned or do not take actions to alleviate their pains. Peltzer et al [6], in their report of pain management among AIDS patients in South Africa, reported that when inquired, a large proportion of patents (87.1%) were using herbal remedies for pain relief.

In a country where the adult HIV sero-prevalence is estimated at about 12% [7], the majority of patients admitted to the adult medical wards at the QECH have HIV infection and other infectious diseases [8-11]. As a result of the high burden of HIV and AIDS in Malawi, there has been concomitant rise in tuberculosis, non-typhoidal salmonella infection, Kaposi's sarcoma, and Cryptococcal meningitis [12-14]. While many of these diseases or medical conditions are associated with pain symptoms, there is limited attention on the quality of care provided to patients. The common often intermediate or long-term end point of patient assessment is survival or mortality, rarely quality of life, such as pain relief.

Pain syndromes are common among AIDS patients [15]. In the 'pre-antiretroviral era', Grant et al reported that relief from pain among the dying was perceived as contributing to a "good death" in rural Kenya [16]. Unlike in 2005 when mortality was almost certain among HIV infected persons admitted to hospitals in Malawi, the situation has currently changed where enhanced chances of survival is possible as a result of widespread availability of antiretroviral therapy [17-19]. The current study was carried out at a period when the "scaling-up" of antiretroviral therapy was just starting in the country [19].

We are unaware of any published studies on the prevalence of pain and the quality of its management among adult medical in-patients in Malawi. We therefore conducted this audit to contribute to literature on the prevalence of pain and its management through an assessment of patients' hospital charts at the QECH in Blantyre, Malawi. Such a report may raise awareness and garner attention towards the study of pain and its management in setting where such an interest is lacking.

## Methods

This was a retrospective, descriptive audit aimed to estimate the prevalence of recorded pain and the quality of its management among adult medical in-patients. The study was carried out at the Queen Elizabeth Central Hospital (QECH), the major referral hospital in Malawi and the main teaching hospital of the University of Malawi, College of Medicine. The hospital is also a teaching center for the Kamuzu College of Nursing (KCN), for training nursing students and the Malawi College of Health Sciences, for the training of clinical officers, medical assistants and enrolled nurses. Like other public referral (and district level) health facilities in Malawi, clinical officers and medical doctors work side by side both in the ambulatory services and within hospital wards.

Consecutive case files for patients who had died or had been discharged within a period of two weeks were included. Data for patients still admitted within the medical wards were not reviewed as there was possibility that their symptoms and management could have changed during the course of the study, and no complete records would be possible as they were still under care. Data were abstracted by two research assistants and a sample verified by one of the investigators. For patients who had been discharged or had died, their reported record of pain or its management had "been sealed." Data were entered into Excel and analysis conduced using SPSS version 9.0 (Chicago, Illinois, United States) to obtain frequencies and correlations. A p value of less than 0.05 was considered statistically significant.

The study was considered an audit and therefore full ethics review board was not done. Permission to conduct the study was provided by the Medical Director of the hospital. The study proposal was also reviewed by Hospice Africa Uganda research committee as it was part of the Diploma in Palliative Care, Makerere University program (Uganda), in which the first author was a student.

## Results

The results are presented below under the following sub-headings: socio-demographic characteristics of patients; prevalence of pain; characterization of pain and recorded management plan.

### Demographic characteristics

A total of 121 patients' case notes were reviewed and analyzed of which 60 (49.6%) and 61 (50.4%) were males and females respectively. Patient's weight was not indicated in any of the files; religion was indicated in only 27 (22.3%) of the files, while 94 (77.7%) had no record of religious affiliation. The majority of the patients, 111 (91.7%%) had their current marital status recorded. A fifth, 21 (17.4%) patients had died while 100 (82.6%) had been discharged alive.

### Prevalence of and sites of pain

Of the 121 patients, 91 (75.2%) were recorded as having at least one pain while 30 patients (24.8%) had no record of pain (including no report of absence of pain). For those patients with recorded pain, the body chart/map was used in 38 (41.8%) cases showing the body site with pain; formal pain severity scores were never used at all. The number of pains was also assessed e.g. a patient with headache, stomachache and backache was labeled as having 3 pains while a patient with only one anatomical site of pain was categorized as having one pain site. The frequency of the different numbers of pain is presented in Table 1 below.

**Table 1:** Number of pain sites in an individual patient among medical in-patients in Blantyre, Malawi.

**Number of pain sites**	**Frequency reported (%)**
0	29 (24.0)
1	51 (53.0)
2	33 (27.3)
3	6 (5.0)
4 or more	1 (0.08)

### Characterization and description of pain

For patients with a record of pain, we aimed to determine the frequency of whether the pain had been characterized. Furthermore we also assessed the description of the pain by recorded by clinicians in the case notes based on severity, periodicity, radiation, alleviating and exacerbating factors. Table 2 provides the results presenting the description studied, whether indicated or not indicated in the case notes.

**Table 2:** Characterization of pain as recorded in patient charts in Blantyre, Malawi.

**Character of pain**	**Frequency recorded (%)**	**Frequency not recorded (%)**
Severity	5 (5.5)	86 (94.5)
Periodicity	43 (47.3)	48 (52.7)
Radiation	7 (7.7)	84 (92.3)
Alleviating factors	5 (5.5)	86 (94.5)
Exacerbating factors	9 (9.9)	82 (90.1)

### Management of pain

Of the 91 patients with history of pain recorded, 88 (96.7%) had recorded previous pain medications received before the current hospital admission. While in hospital for the current hospitalization however, analgesics were prescribed for 70/91 (76.9%), the commonest of which was paracetamol 62 (68.1%), aspirin 9 (9.9%), while other analgesics and opioids were prescribed less than 3%. The majority of patients (67.8%) were prescribed as thrice daily intake of analgesics. Drug adjuvant treatment was prescribed for only 2 patients. 31 (34.1%) patients were first seen by doctors while the rest had been first seen by other clinical staff such as clinical officers. Males with pain were 3.2 times more likely to receive analgesics as compared to female patients ((p < 0.01).

We also aimed to determine whether clinicians were interested to follow up on pain among the patients. This was assessed by identifying whether any record on pain was made at second patient review following admission. At second clinician consultation, only 8 (8.8%) of the 91 patients had any information on pain while the rest 83 (91.2%%) had no record of the pain on a second ward consultation. The quality of sleep among patients was recorded in only one patient and in 19 (20.9%) of patients with pain on admission it was reported they had pain on discharge or the last consultation prior to death.

## Discussion

The prevalence of reported pain among adult in-patients at QECH, Blantyre was 75.2%; this was much higher than the 54% pain prevalence reported by Dix et al [20] among medical in-patients in the UK. The majority of patients in our study were possibly inadequately managed with regard to their pain. This conclusion is reached when we assume that what was recorded was what was asked and done to the patients.

Furthermore, it appeared that clinicians did not care to ask about associated symptoms with the pain such as interference of sleep [21]. In our study, assessment of sleep interference was only recorded for one patient.

In both pre-service and in-service training of clinical personnel, adequate history taking and physical examination are emphasized as important tools for adequate diagnosis and management of patient symptoms. The adequate documentation and management of pain symptom is clinical practice should therefore expected. Soyannwo and Amanor-Boadu, [22] however reported that among 44 specialist surgeons, only 18% knew about the WHO's management guidelines for cancer pain by the clock [23]. For adequate management of pain, there is need to ask patients and document the periodicity, severity, character of pain such as nature of pain e.g. throbbing, stabbing, pricking, aching and alleviating and exacerbating factors. Assessment of pain enables the clinician to diagnose the possible aetiology of the pain as either somatic or neuropathic and effectively manage it [24].

The WHO analgesic ladder when used properly is likely to lead the clinicians to provide analgesic(s) and adjuvant therapy based on the characterization and severity of the pain.

For instance, noting about the periodicity of pain (continuous, episodic), severity and sleep disturbance, will enable the clinician determine the functional disturbance the pain may be causing the patient.

It is a matter of concern that the majority of patients in our study did not have important socio-demographic data recorded, for instance, religion. To provide holistic care, there is need to have an indication of the religious or spiritual beliefs of the patient. Knowledge of spiritual beliefs for instance will enable the care team to facilitate linkages with spiritual leaders, especially as many of our patients are terminally ill. Similarly, upon death, it is important to be aware of any religious requirements for preparing the body for keeping or burial according to the patient's religious beliefs [25,26].

While the other socio-demographic characteristics were not adequately recorded, it is important to note that marital status was recorded 92.4% of the time. Knowledge about the patients' marital status will enable the care team to identify the 'significant' others in the patient's life. Decisions on the pattern of care when discharged may also have to be discussed with family member, if available.

The reasons why males with pain were more likely to receive analgesics as compared to females with pain can not be deduced from the present study. It is possible that male patients were better able to communicate with clinicians (who in this setting are likely to be male). Paracetamol was the most commonly prescribed analgesic followed by aspirin. These are the commonest available analgesic at QECH and this may explain clinician's preferences to prescribing these drugs. On the other hand, lack of appreciation of the analgesic ladder and poor assessment and recording could lead clinicians to favour first level analgesics as prescribing the higher levels and adjuvants really means that one understands the characterization of the pain.

Our study had a number of limitations. Firstly, we assumed that if any piece of information was not available in the case notes, we decided that the relevant questions were never asked and the health professional who took the patient's history did not have the information. In actual practice, this may not be the case. Secondly, our audit may not have collected data from a representative sample of patients attending medical wards. However, we decided to collect case files of patients who had "passed through" the wards over a two week period. If there had been changes in the patient profiles or clinical staff, the sample studied may not have been representative. Thirdly, we only studied patients in the medical wards. Our findings may therefore not be representative of the diversity of patients attending the hospital. Finally, the clinician case load and other work environment situations were not assessed so as to explain the observed quality of management.

## Conclusion

The present study was unable to determine why clinicians at QECH were unable to document pain adequately in patients' case notes. While this study is primarily of local focus, we believe that our findings can spur research and consideration of adequate of management of pain in similar setting where it has not received proper attention. We suggest that the factors that prevent clinicians from adequately manage pain be further studied in a prospective study. Possibilities for the current practice include; lack of adequate time to document pain as many of wards at QECH have few staff attending many patients, lack of appreciation by clinicians on proper management of pain, especially using the WHO's analgesic ladder when indicated. This is particularly worrying for QECH which is the teaching hospital for medical students and nurse trainees. Lack of models among trainees may impact negatively on proper pain management in generations to come. There is need to explore via operational research whether pain protocols as suggested by Beck [27] may eventually contribute to adequate pain management in Malawi.

## Competing interests

The authors declare that they have no competing interests.

## Authors' contributions

ASM designed the study, supervised research assistants in abstracting data from patients' case note and participated in the interpretation of the findings and drafting of the manuscript. HEM analysed the data and participated in the interpretation of the findings and drafting of the manuscript. All authors read and approved the final manuscript.
